# A new genus of soft coral of the family Alcyoniidae (Cnidaria, Octocorallia) with re-description of a new combination and description of a new species

**DOI:** 10.3897/zookeys.84.781

**Published:** 2011-03-01

**Authors:** Yehuda Benayahu, Catherine S. McFadden

**Affiliations:** Department of Zoology, George S. Wise Faculty of Life Sciences, Tel Aviv University, Ramat Aviv, Tel Aviv 69978, Israel; Department of Biology, Harvey Mudd College, Claremont, CA 91711-5990, USA

**Keywords:** Alcyonacea, South Africa, Kenya, Gulf of Oman, Taiwan, Japan, coral reefs

## Abstract

A new genus, Aldersladum (family Alcyoniidae), is established to accommodate a previously described species, Efflatounaria sodwanae Benayahu, 1993 (family Xeniidae) from Sodwana Bay, South Africa that was wrongly assigned to the latter genus. This species is redescribed and a second new species, Aldersladum jengi from Penghu Is., Taiwan, is described. The diagnostic features of the new genus include the presence of only figure-eight shaped platelets in all parts of the colony, thus differentiating it from all known genera of the Alcyoniidae. Based on examination of additional material from other localities, the zoogeographical distribution of the genus is confirmed to include the coral reefs of South Africa, Kenya, Gulf of Oman, Taiwan and Japan. Phylogenetic analyses of two mitochondrial genes strongly support its placement in the family Alcyoniidae.

## Introduction

In a study of Alcyonacea from Sodwana Bay, South Africa, the new species, Efflatounaria sodwanae Benayahu, 1993, (family Xeniidae) was described. This species features a low crust and has platelet-like sclerites with a distinct waist and either single or double pits on their flat surface, thus resembling a figure-eight. During an octocoral survey conducted in 2006 on the reefs of Penghu Is., Taiwan, a colony with finger-like, flabby lobes was collected and found to have a similar type of sclerite to those of Efflatounaria sodwanae. While comparing the colonies of South Africa and Taiwan, we were intrigued by their figure-eight sclerites, which resembled those found in polyps of the genera Cladiella Gray, 1869, Klyxum Alderslade, 2000 and Rhytisma Alderslade, 2000 of the family Alcyoniidae (see Fabricius and Alderslade 2001), yet had never been recorded among genera of the family Xeniidae (e.g., Alderslade 2001; Benayahu in press). These findings led us to thoroughly re-examine the type material of Efflatounaria sodwanae, also in relation to the colony from Penghu Is. and other related material kept at the Zoological Museum of Tel Aviv University (ZMTAU) and at the Netherlands Center for Biodiversity, Naturalis, formerly Rijksmuseum van Natuurlijke Historie, Leiden (RMNH). The findings resulted in establishing Aldersladum gen. n. (family Alcyoniidae) for placement of the above-mentioned material, which includes Aldersladum sodwanum comb. n., which is also re-described; and Aldersladum jengi sp. n. which is depicted and described. Based also on molecular analyses, the phylogenetic position of the new genus is discussed.

## Materials and methods

The material examined in this study was obtained during several octocoral surveys conducted in north-east Taiwan (1998); Yoron Is. and Okinoerbu Is., Ryukyu Archipelago, Japan (2000); Kenya (2001, 2002 and 2003); Penghu Is., Taiwan (2006) and the Gulf of Oman (2009). The 1998 and 2000 material was initially fixed in 4% formalin in seawater, rinsed in fresh water after 24 hours, and then stored in 70% alcohol. The later collections, from 2001 onwards, were fixed in 70% alcohol and subsamples were removed and preserved in absolute alcohol or DMSO for molecular studies. Sclerites from different parts of the colony (polyps, polypary, base-surface and interior) were obtained by dissolving the tissues in 10% sodium hypochlorite, followed by careful rinsing in fresh water. They were then prepared for scanning electron microscopy as follows: the sclerites were carefully rinsed with double-distilled water, dried at room temperature, coated with gold and examined with a Jeol 840A electron microscope, operated at 15 kV. The identified specimens are deposited at ZMTAU and at RMNH as indicated below.

## Taxonomy

### Aldersladum gen. n.

urn:lsid:zoobank.org:act:43D3F04F-EE2D-44AD-9D0B-4EC510F0B1A6

#### Type species.

Efflatounaria sodwanae Benayahu, 1993: 11–14, here designated.

#### Etymology.

The genus name (gender-neutral) honors Dr. Phil Alderslade, a prominent octocoral taxonomist and a friend, in recognition of his immense contribution to the study of octocorals.

#### Diagnosis and description.

Colonies have a low encrusting base, holdfast-like, from which lobes arise. The lobes vary in size from short, knob-like to longer, finger-like. The non-retractile monomorphic polyps are densely arranged on the lobes. The same type of sclerite is found in all parts of the colony, as confirmed by both light microscopy and SEM examination. It comprises platelets that are narrower across their mid-lateral line. A longitudinal slit, sometimes narrower in its middle part, is commonly found on the platelet’s flat surface, located at the center and occupying about half its length, thus giving the sclerite a typical figure-eight form. The surface of the platelets is characterized by the appearance of an uneven crystal deposition that gives it a porous texture. Colonies are zooxanthellate.

#### Aldersladum sodwanum

(Benayahu, 1993) comb. n.

Figs 1 2 6a

Efflatounaria sodwanae Benayahu 1993: 11–14.

##### Holotype and 2 microscopic slides:

ZMTAU Co 27902, South Africa, Sodwana Bay, Nine-mile Reef, 23 July 1992, 16 m 1992, leg. Y. Benayahu. Paratypes: ZMTAU Co 27933 and 27935 details as above; ZMTAU Co 27934, South Africa, Sodwana Bay, Nine-mile Reef, 5 May 1992, 10 m, leg. M.H. Schleyer. Other material: ZMTAU Co 30465, Kenya, Mombassa, off Likoni (Wall reef), 04°06'S, 39°41'E, 20–22 m, 27 January 2000; ZMTAU Co 31165, Kenya, Mombassa, off Likoni, Shelly Beach, 04°07'S, 39°40'E, 20–26 m, 20 February, 2001; ZMTAU Co 31520, Kenya, Mombassa, off Likoni, Shelly Beach (Turning Buoy), 04°05'S, 39°41'E, 15–28 m, 27 February 2002; ZMTAU Co 31598, Kenya, Wasini Is., Shimoni Channel, 04°00'S, 39°22'E 8 m, 7 March 2002; ZMTAU Co 32579, Kenya, Kilifi (Mooring), 03°58S, 39°46'E. All other material listed above was collected by Y. Benayahu and S. Perkol-Finkel. ZMTAU Co 31092, Aoti, north-east coast of Taiwan, 7 July 1998, Coll. M.-S. Jeng; RMNH Coel. 39925, Gulf of Oman, Iran, Chabahar, 25°16'29.251"N, 60°40'32.189"E, 3 m depth, exposed rocky substrate, leg. K. Samimi-Namin, 27 January 2009. Each ZMTAU Co and RMNH Coel number represents one colony.

**Figure 1.**
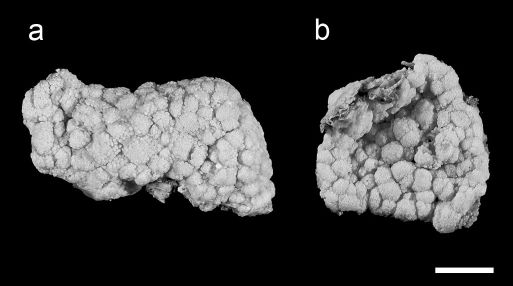
Fixed specimens of Aldersladum sodwanum comb. n.; **a** holotype ZMTAU Co 27902 **b** 27934 paratype. Scale = 10 mm.

##### Diagnosis and description.

For the sake of convenience the revised description also contains the relevant information that appeared in the original description of Efflatounaria sodwanae. The holotype has a firm, low, crust-like base, 3–5 mm high, attached to a calcareous fragment. The maximum cross-section of the colony is 6 × 3 cm, and its total height (base and polypary) is up to 8 mm (Fig. 1a). The polypary consists of numerous knob-like lobes, some of which bud off into one to three smaller side lobules. Obscure material, composed of slime and debris, is found between adjacent lobes. The polyps are confined only to the lobes and are absent from their basal part. The anthocodial wall of some polyps is partially contracted and, very rarely, the tentacles are withdrawn into the mouth.

**Figure 2.**
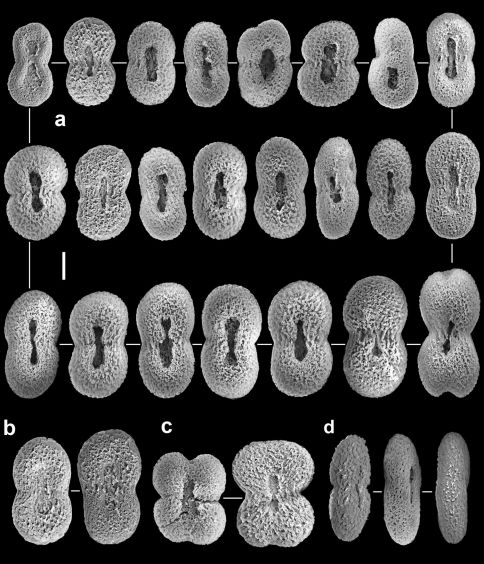
Aldersladum sodwanum comb. n., holotype ZMTAU Co 27902; figure-eight sclerites; **a** with median slit **b** with poorly developed median slit **c** wide sclerites **d** lateral view. Scale = 0.010 mm, that at **a** also applies to **b–d**.

The sclerites are platelets of porous texture, narrower across the lateral middle-line, 0.032–0.048 mm long (Fig. 2). They are found in the polyps and in all parts of the colony. A longitudinal median slit on the flat surface of the sclerite occupies about half of its length. The slit can be narrower in its middle and occasionally widens at its ends, forming aperture-like structures (Fig. 2a). Some of the sclerites have a poorly developed slit (Fig. 2b) while in others this is wider (Fig. 2c), but both possess similar common features. The architectural features of the sclerites confer upon them a figure-eight shape. Under a light microscope the slit and apertures are observed on the surface of the sclerite as bright median areas. Although some sclerites appear as cigar-shaped, these are actually platelets viewed from their narrow lateral surface, as also confirmed by scanning electron microscopy (Fig. 2d). The alcohol preserved colony is cream-beige.The preserved paratypes differ in size (e.g., Fig. 1b).

When alive the polyps are light brown and the base of the colony is brighter (Fig. 6a). The colonies are quite small, commonly no larger than 30–40 mm in diameter.

#### Aldersladum jengi sp. n.

urn:lsid:zoobank.org:act:DDCC6193-547A-4DD7-A62A-A570461AFBCA

**Figure 3.**
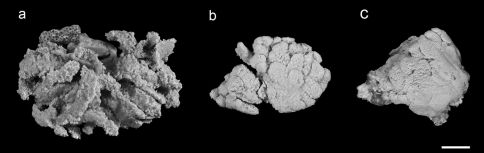
Fixed specimens of Aldersladum jengi gen. n. and sp. n.; **a** holotypeZMTAU Co 33607 **b** paratype ZMTAU Co 31687 **c** paratype ZMTAU Co 31688. Scale = 10 mm.

Figs 3 [Fig F4] [Fig F5] 6b 

##### Holotype:

ZMTAU Co 33607 and 2 microscope slides, Taiwan, Penghu Is. Gupo Reef, 23°42'859"N, 119°33'488"E; 2–8 m, 29 July 2006. Paratypes: ZMTAU Co 31687, Japan, Ryukyu Archipelago, Yoron Is., 27°03'28"N, 128°23'59"E, 9 m, 1 July 2000; RMNH Coel. 39926, Japan, Ryukyu Archipelago, Okinoerabu Is. 27°23'38"N, 128°31'32"E, 11 m, 30 June 2000. Each ZMTAU Co number represents one colony. Material was collected by Y. Benayahu.

##### Etymology.

The species is named after Prof. M.-S. Jeng, Institute of Zoology, Academia Sinica, Taipei, Taiwan, in appreciation of his continuous support of octocoral studies in Taiwan.

##### Diagnosis and description.

The holotype is a flabby colony with a maximum cross-section of 6 × 5 cm (Fig. 3a). It has a low base, holdfast-like, 3–5 mm high, attached to a fragment of calcareous substrate. The base gives rise to primary finger-like lobes that occasionally branch once or twice into short lobules. The polyps feature densely on the lobes, with their density markedly decreasing on the basal part. The polyps are expanded and in only a few of them the anthocodial wall is partially contracted. Very rarely, the tentacles are withdrawn into the mouth.

**Figure 4. F4:**
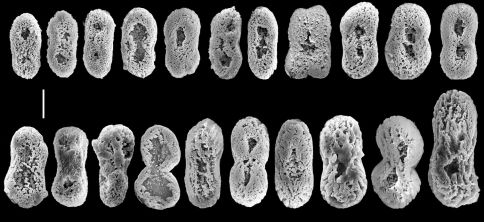
Aldersladum jengi gen. n. and sp. n., holotype ZMTAU Co 33607; figure-eight sclerites of the polyps and the lobules. Scale = 0.010 mm.

**Figure 5. F5:**
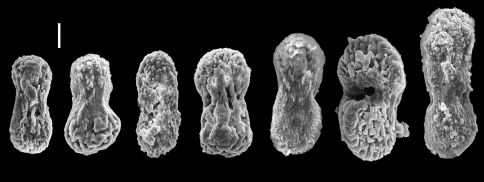
Aldersladum jengi gen. n. and sp. n., holotype ZMTAU Co 33607; rods with median constriction from colony base. Scale = 0.010 mm.

The sclerites are platelets, mostly narrower across their lateral middle-line, 0.023–0.042 mm long (Fig. 4). These sclerites are found in all parts of the colony, including the polyps. The vast majority of sclerites have a longitudinal median slit on their flat surface. The slit is often calcified along most of its length, thus leaving two, or rarely one, aperture-like structures at its ends. The base of the colony has fewer sclerites compared to the lobes; these reach up to 0.060 mm long, mostly with no median slit (Fig. 5). These latter sclerites may have a circumferential waist, rather than being narrow across their lateral middle-line. The architectural features of these sclerites give them the appearance of rods with a median constriction (Fig. 5), which is more pronounced compared to the sclerites of the lobes (Fig. 4). All sclerites feature a “spongy” texture that seems to result from the uneven alignment of the crystal-nodules that construct them. The colony is zooxanthellate. The alcohol- preserved colony is cream-beige.

The preserved paratypes differ in size (Fig. 3b, c) and are more rigid than the holotype. The few sclerites in the base of both paratypes are up to 0.080 mm long, and thus larger compared to those found in the holotype.

**Figure 6.**
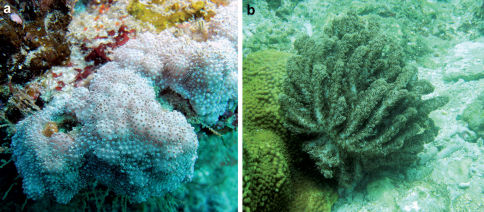
Underwater photographs of **a** Aldersladum sodwanum comb. n.* **b** holotype of Aldersladum jengi gen. n. and sp. n. ZMTAU Co 33607. *Courtesy of M.H. Schleyer.

When alive the polyps of the holotype were dark brown and the surface of the lobes was brighter (Fig. 6b). The flabby lobes tend to undulate with the water surge and in this sense resemble certain Klyxum colonies (pers. obs.)

## Discussion

The generic diagnosis of Efflatounaria is based on Efflatounaria tottoni Gohar, 1939, a species that lacks sclerites and is characterized by retractile polyps. Fabricius and Alderslade (2001: 144–145) noted that in this genus “They [polyps] are so highly contractile that they can deflate until flush with the colony surface and appear to be retracted”. To the best of our knowledge, except for the original description, the literature has since only once mentioned the occurrence of Efflatounaria tottoni (Okinawa: Benayahu 1995: 108, 124). The latter colony has the suite of diagnostic features noted by Gohar (1939), including the typical colony shape, retractile polyps and absence of sclerites. Interestingly, Gohar (1939) assigned Cespitularia mantoni Hickson, 1931 to Efflatounaria and as a result included a species with sclerites in this genus. The lack of sclerites is therefore not an obligate feature of the genus. Both genera feature the corpuscle-like platelets or spheroids that are typical of other members of the Xeniidae. It should also be noted that at present there is some ambiguity concerning the morphological distinction between the two xeniid genera, Efflatounaria and Cespitularia Milne-Edwards & Haime, 1850 (see Fabricius and Alderslade 2001), which further complicates the placement of species in these two genera.

Re-examination of the holotype of Efflatounaria sodwanae (see also below) revealed no real sign of either contractile or retractile polyps. Indeed, in some of the polyps the anthocodial wall is contracted (Benayahu 1993: 11) to a limited degree. The sclerite-architecture of Efflatounaria sodwanae (Benayahu 1993: 13) markedly differs from the corpuscle-like platelets or spheroids attributed to xeniids (see Fabricius and Alderslade 2001). Based on these features it is evident that Efflatounaria sodwanae can not retain its original generic placement.

The following genera of the family Alcyoniidae – Cladiella Gray, 1869; Klyxum Alderslade, 2000 and Rhytisma Alderslade, 2000 – have figure-eight sclerites resembling those of Aldersladum gen. n. However, in these genera they are confined only to the polyps, while other parts of the colony feature different diagnostic sclerites (Cladiella: dumbbells, Klyxum: spindles and granular rods, Rhytisma: spindles; i.e., Fabricius and Alderslade 2001). We suggest, therefore, that the presence of figure-eight sclerites in all parts of the colony justifies the establishment of Aldersladum gen. n. for placement of Efflatounaria sodwanae. Since this type of sclerite occurs only in the family Alcyoniidae, the new genus is assigned to that family. Preliminary molecular data based on mitochondrial *msh1* and COI gene sequences strongly support the placement of Aldersladum in a clade with the alcyoniid genera Klyxum and Cladiella (McFadden unpub. data).

The current study expands the zoogeographical distribution of Aldersladum sodwanum from the type locality (Sodwana Bay, South Africa) to several sites along the Kenyan coast, from Likoni, off Mombassa in the north, to Wasini Is. in the south. This species has also been recorded from the Gulf of Oman and north-east Taiwan (see above). It is therefore evident that Aldersladum sodwanum features a wide zoogeographical distribution ranging from the East-African coast to the Pacific reefs. We suggest that the infrequent appearance of the species on the reefs may have hindered its record in reef surveys.

Aldersladum jengi fits the diagnosis of Aldersladum gen. n. by having lobes arising from a narrow base, non-retractile polyps and figure-eight sclerites found in all parts of the colony. Its lobes are much longer compared to Aldersladum sodwanum. The porous sclerite texture of Aldersladum jengi is coarser compared to that of its congener (Fig. 2 vs. Fig. 4, 5). The sclerites of the colony base are longer, with some featuring a circumferential waist. We consider these differences sufficient to justify the separation between the two species and to establish Aldersladum jengi as a second species within the genus. Analysis of molecular data (mitochondrial *msh1* and *COI* genes) also supports the genetic distinction between these two species; the pair-wise genetic distance (uncorrected p) between Aldersladum sodwanum and Aldersladum jengi is 0.4%, comparable to or greater than that observed among some morphospecies in the closely related genera Klyxum and Cladiella (McFadden et al. 2011; unpub. data).

## Conclusions

Among the genera of the family Alcyoniidae the genus Aldersladum seems to be the only one to possess figure-eight sclerites in all parts of the colony. In the alcyoniids Cladiella, Klyxum and Rhytisma this type of sclerite is found only in the polyps, and different, diagnostic sclerite-forms characterize other parts of the colony. Indeed, no other genus of Alcyoniidae has only a single sclerite form in all regions of the colony; for example, Sinularia May, 1898 has clubs on the surface of the lobes and base but spindles in its interior (see Fabricius and Alderslade 2001). Although some species of the genera Xenia and Ovabunda (family Xeniidae) also have uniform sclerites in all parts of the colony (Benayahu, in press), they are not figure-eights. The xeniid genera Ingotia, Ixion and Bayerxenia have platelet-like sclerites that are narrow across the lateral middle-line like those of Aldersladum, but they lack the median slit that gives the figure-eight appearance; moreover, they are not the only sclerite-form found throughout the colony (Alderslade 2001). Sclerites of Moolabalia (family Clavulariidae) also lack the median slit, and the stoloniferous growth form of this genus is distinctive (Alderslade 2001). Placement of the newly-assigned genus Aldersladum in the family Alcyoniidae on the basis of sclerite- form is supported by molecular results that clearly show that Aldersladum is a close relative of Cladiella and Klyxum.

In the original description of Efflatounaria sodwanae the resemblance between the fine structure of its sclerites and those of Cladiella daphnae Ofwegen & Benayahu, 1992 was noted, indications that its significance in the systematics of Alcyoniidae needed to be studied (Benayahu 1993). There is a certain similarity between Aldersladum jengi and Cladiella daphnae as reflected in their flabby lobes, figure-eight sclerites and the resemblance between the base sclerites of the former (Fig. 5) and the surface stalk sclerites of the latter (Ofwegen and Benayahu 1992: Fig. 2 i-n). Despite this similarity, it should be noted that Cladiella daphnae features different sclerites in the lobes to those in the base (Ofwegen and Benayahu 1992: Figs 2, 3: figure-eights vs. dumbbells) and hence it differs from both Aldersladum sodwanae and Aldersladum jengi.

## Supplementary Material

XML Treatment for Aldersladum
